# Measuring microtubule dynamics

**DOI:** 10.1042/EBC20180035

**Published:** 2018-10-04

**Authors:** Alexander James Zwetsloot, Gokhan Tut, Anne Straube

**Affiliations:** 1 Centre for Mechanochemical Cell Biology, University of Warwick, Coventry, CV4 7AL, U.K.; 2 MRC Doctoral Training Partnership, University of Warwick, Coventry, CV4 7AL, U.K.; 3 Division of Biomedical Sciences, Warwick Medical School, Coventry, CV4 7AL, U.K.

**Keywords:** dynamic instability, EB3, kymograph, microtubules, +TIPs, tubulin

## Abstract

Microtubules are key players in cellular self-organization, acting as structural scaffolds, cellular highways, force generators and signalling platforms. Microtubules are polar filaments that undergo dynamic instability, i.e. transition between phases of growth and shrinkage. This allows microtubules to explore the inner space of the cell, generate pushing and pulling forces and remodel themselves into arrays with different geometry and function such as the mitotic spindle. To do this, eukaryotic cells employ an arsenal of regulatory proteins to control microtubule dynamics spatially and temporally. Plants and microorganisms have developed secondary metabolites that perturb microtubule dynamics, many of which are in active use as cancer chemotherapeutics and anti-inflammatory drugs. Here, we summarize the methods used to visualize microtubules and to measure the parameters of dynamic instability to study both microtubule regulatory proteins and the action of small molecules interfering with microtubule assembly and/or disassembly.

## Why measure microtubule dynamics?

**Figure 1:**
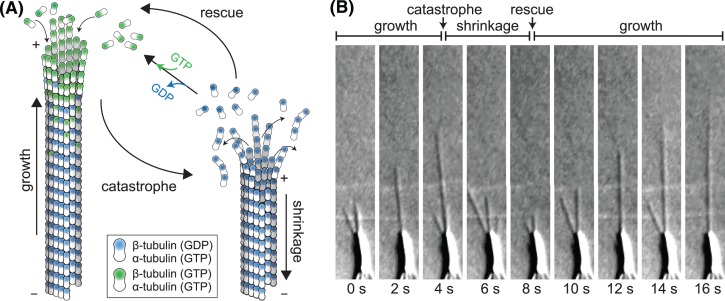
Microtubule dynamic instability (**A**) Microtubules are tubular filaments assembled from αβ-tubulin heterodimers arranged head-to-tail in 13 helically arranged protofilaments. Microtubules grow by the addition of GTP-tubulin (green) to their ends. GTP hydrolysis occurs when β-tubulin is buried in the lattice. Catastrophe, i.e. transition to shrinkage occurs when the GTP cap is lost and GDP-tubulin is exposed at the microtubule end. The transition from shrinkage to growth is called rescue. Note that α-tubulin contains a non-exchangeable GTP trapped at the intradimer interface. (**B**) DIC images from a time series of microtubules growing from an axoneme (large object at bottom of image). Imaging data courtesy of Douglas Drummond and Rob Cross. Abbreviation: DIC, differential interference contrast.

One of the most striking properties of microtubules is their dynamic instability (Figure 1). Extensive phases of microtubule growth are followed by rapid disassembly and regrowth [1]. Microtubules grow by the addition of GTP-bound αβ-tubulin heterodimers to their ends. Incorporation into the microtubule facilitates GTP hydrolysis, resulting in the growing tip being capped by GTP-tubulin while the microtubule lattice consists primarily of GDP-tubulin. Exposing GDP-tubulin at the tip results in catastrophe, the switch from growth to shrinkage. Microtubule dynamics, therefore, are driven by the delicate balance of GTP-tubulin incorporation and rate of GTP hydrolysis [2].

Dynamic instability enables the formation of long filaments that can be reorganized on the timescale of minutes. For example, at the onset of mitosis, interphase microtubules are disassembled and new microtubules form the bipolar mitotic spindle, capture, align and then separate chromosomes by making dynamic linkages to the kinetochore [3,4]. Also in non-dividing cells, microtubules perform important functions. For example, neuronal cells maintain polarized microtubule bundles in axons and dendrites for the duration of an animal’s life, thereby allowing the directional transport of cargoes by kinesins and dynein [5,6]. Similarly, muscle cells arrange microtubules paraxially to provide structural integrity and resistance to contractile forces [7,8]. In motile cells, microtubules control directionality of cell locomotion in a crosstalk with the actin cytoskeleton and by mediating the turnover of adhesion sites [9]. Thus, proper microtubule arrangement and the spatiotemporal control of microtubule dynamics are integrally important to cell morphology, function and their ability to faithfully proliferate. Due to their ability to interfere with microtubule dynamics and thus inhibit cell proliferation, microtubule-targeting agents such as taxanes and vinca alkaloids are widely used in the clinic as cancer chemotherapeutics [10,11].

To understand how the cellular machinery regulates microtubule dynamics and to reveal the mechanism by which small molecule inhibitors stabilize or destabilize microtubules, we need to quantitatively investigate the microtubule cytoskeleton. This can be done by reconstituting microtubule dynamics from purified tubulin *in vitro*, and is most precisely measured by using imaging-based assays to record the behaviour of individual microtubules and the direct effect of any added protein or chemical. *In vitro* experiments usually permit measurement of all four parameters of dynamic instability (growth, shrinkage speeds, catastrophe and rescue frequencies). Additional information on nucleation frequency, flexural rigidity or tubulin turnover in the microtubule shaft are also accessible in such reconstitution experiments. Alternatively, or additionally, dynamic microtubules can be studied inside living cells. In many cellular systems, the high density of microtubules poses challenges to observing individual microtubules and thus to measuring all parameters of microtubule instability. Therefore, a number of complementary approaches and markers might need to be used to glean information about the dynamic state of microtubules. Here, we review the current approaches in the field to investigate microtubule dynamics both in reconstitution experiments as well as in cells.

## Measuring microtubule dynamics *in vitro*

A simple way to measure microtubule assembly is to measure the turbidity of a solution of soluble tubulin upon the addition of GTP as the forming microtubules scatter the light roughly proportionally to their mass [12,13]. Such bulk measurements allow some insight into whether a compound has a stabilizing or destabilizing effect on microtubules, and can be performed at high throughput. Without any addition of labels, individual microtubules can be visualized using darkfield or differential interference contrast (DIC) microscopy (Figure 1B) and their growth and shrinkage speeds as well as transition rates measured from timelapse images [14,15]. Darkfield or DIC microscopy are still the methods of choice if working with limiting sources of tubulin such as single isoform tubulin preparations [16,17], or to exclude effects from fluorescent labels [18]. However, the availability of both commercial imaging systems and fluorescently labelled tubulin, and the ability to study the dynamic relocalization of the protein-of-interest at the same time makes total internal reflection fluorescence (TIRF) microscopy currently the most popular method for studying microtubule dynamics *in vitro*.

**Figure 2:**
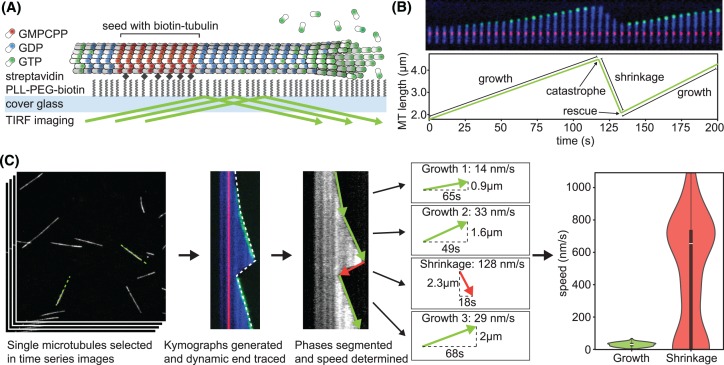
Microtubule dynamics measurement *in vitro* (**A**) Typical TIRF-based assay to reconstitute and measure microtubule dynamics *in vitro* in which a GMPCPP-stabilized microtubule seed is immobilized on a passivated glass surface that nucleates a microtubule. The seed and free tubulin typically contain 5–20% tubulin that is labelled with different fluorophores. Microtubule-binding proteins or small molecules can be added and their binding as well as effect on microtubule dynamics measured in this assay. (**B**) Montage and life history plot of a dynamic microtubule with seed (red), dynamic microtubule lattice (blue) and microtubule tip labelled with EB3-GFP (green). Phases are labelled as growth and shrinkage, transitions marked as catastrophe and rescue. (**C**) Workflow to measure microtubule dynamics parameters including the generation of kymographs (space–time plots), automatic or manual detection of the edge and extraction of speed and transition rates. The final violin plots are vertical histograms that show the full distribution of growth and shrinkage speeds that make up the median, which is also depicted by an inset boxplot.

Typically, dynamic microtubules are assembled from a template (Figure 2A). This can either be purified centrosomes that are a source of γ-tubulin ring complexes from which new microtubule growth may be nucleated or axonemes that contain microtubules, which can be extended on [19,20]. More commonly, short stable microtubule seeds are polymerized using the non-hydrolyzable GTP analogue GMPCPP. Seeds are attached to the coverslip either using antibodies against a fluorophore or via streptavidin linking to biotin–tubulin. The specific immobilization of the template and passivation of the remaining coverslip surface result in the dynamic microtubule being free of, but in close proximity to, the surface thereby allowing TIRF microscopy [21]. Growing microtubules can be observed either through the addition of a few percent fluorescently labelled tubulin within the reaction mixture or by fluorescently labelled microtubule-associated proteins (MAPs) such as EB3 [22]. Imaging over a period of several minutes allows the observation of growth and shrinkage of microtubules (Figure 2B). To extract the parameters of dynamic instability, the microtubule ends can be tracked directly in the timelapse movie, but it is more common to generate kymographs and trace the path of the microtubule end either manually or using automatic image analysis tools (Figure 2C). For example, the automatic extraction of microtubule dynamics parameters from kymographs of DIC images, is possible if using optimized filtering and edge detection algorithms [23]. Gaussian models have been fitted directly to fluorescence images of microtubule to detect microtubule motility, but also depolymerization speeds with nanometre precision [24]. However, to determine the end position in microtubule dynamics assays with immobilized seeds, the direct fitting of a 2D Gauss error function to the microtubule end is sufficient to detect length changes at subpixel resolution. The variance of the Gauss error function can also reveal the extent of taper at the microtubule end, i.e. the difference between the leading and lagging protofilaments [25]. All direct fitting approaches depend on high signal–noise ratio (SNR) [26] and will only result in meaningful data on the microtubule tip structure if a large proportion (>25%) of fluorescently labelled tubulin is used (Fitton & Straube, unpublished data). Finally, automatically or manually derived tracking data are used to calculate speeds and transition rates (Figure 2C). For that purpose, the catastrophe rate is defined as the number of growth-to-shrinkage transitions divided by the total time microtubules spent growing and inversely, the rescue frequency is calculated as the number of events when a shrinking microtubule resumes growth before reaching the seed divided by the total time spent shrinking.

While catastrophe frequencies are thought to be inversely proportional to the stability of the GTP cap, a more direct approach to determining GTP cap size and stability is to rapidly remove the supply of free tubulin and measure the time to catastrophe [27,28]. The finding that the measured GTP cap lifetime correlates very closely to the intensity of the EB1 comet at the moment of buffer exchange [28] suggests that in order to assess GTP cap stability, EB comet size could be measured instead. The only caveat to this approach is that EB1 itself is changing the GTP cap and might accelerate GTP hydrolysis [29].

Finally, microtubules experience resisting forces when assembling inside cells. This can be mimicked *in vitro* by growing microtubules into barriers. Such experiments demonstrate that microtubule polymerization speed and catastrophe frequency are strongly force dependent [30]. It is important to study the force dependence as these effects could be further exacerbated in the presence of microtubule regulators that accelerate growth by assembling elaborate tip structures, which are likely to be unstable under force. Measuring microtubule dynamics under force also enables very precise measurements of nanoscale length changes. To do this, the force is kept constantly low and the displacements of a bead attached to a microtubule seed or axoneme are used to derive nanoscale microtubule length measurements [31,32]. Such nanoscale measurements can reveal changes in the fluctuations during a growth phase providing insights into the mechanism of microtubule regulators.

While reconstitution experiments are powerful to reveal the direct effect of a protein or small molecule on microtubule assembly and disassembly, the complex interaction of lattice and tip binding microtubule regulators and spatial constraints that together bring about spatiotemporally regulated microtubule dynamics in cells is difficult to reconstitute. A bridge between the reductionism of *in vitro* reconstitution and complexity of in cell experiments can be achieved with the use of cell extracts. Xenopus egg extracts have been used extensively over the years to study microtubule dynamics, nucleation and spindle formation. Microtubules are visualized by addition of fluorescently labelled tubulin to the extracts, while extraneous nucleation can be induced by the addition of purified centrosomes [33]. Individual proteins can be depleted from the extract using specific antibodies and labelled components can be titrated into the extract for localization or to study concentration-dependence [34–36].

To gain a more holistic view of how a drug or protein affects microtubule dynamics, mechanistic understandings gained from *in vitro* experimentation must be contextualized inside cells, and conversely, phenotypes observed in cells must be understood mechanistically *in vitro*. For this reason, many people complement *in vitro* research with cellular studies.

## Observing microtubule dynamics in cells

Measuring microtubule dynamic instability in cells requires being able to visualize individual microtubules. While it is possible to see microtubules without any labels in thin sections of the cell using video-enhanced differential interference contrast microscopy [37,38], fluorescent techniques either using the injection of chemically labelled tubulin [39,40], cell permeable dyes [41] or DNA-encoded fluorescent protein fusions allow, in principle, to image every microtubule in the cell. However, due to the density of microtubule arrays in many cell types and in particular within the mitotic spindle, it is often not possible to observe individual microtubules for their entire life history and thus obtain all parameters of dynamic instability from a single experiment as it is possible in the reconstitution experiments described above. Thus different labelling techniques are used to obtain insights into distinct aspects of microtubule dynamics.

**Figure 3:**
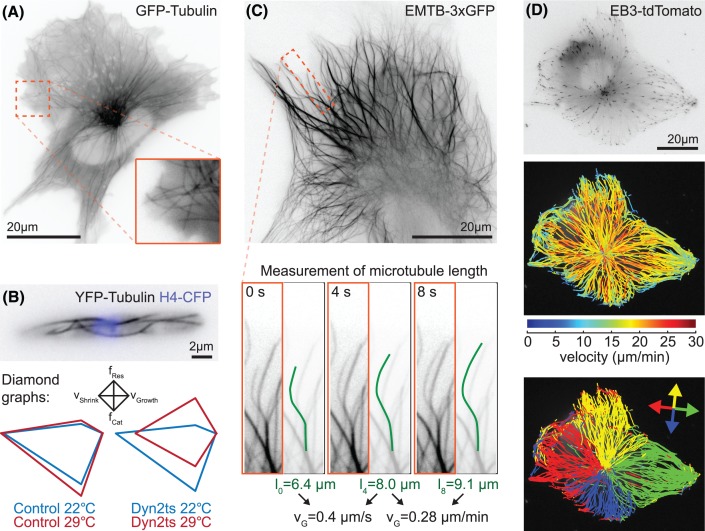
Microtubule dynamics measurement in cells (**A**) Widefield image of a human hTERT RPE1 cell expressing GFP-tubulin. Individual microtubules can be observed, especially close to the cell cortex (inset). (**B**) Widefield image of *Ustilago maydis* cell expressing YFP-tubulin (grey) and histone4-CFP (blue). Individual microtubules are visible throughout the cell, enabling measurement of four parameters of dynamics instability. Diamond graphs show growth speed (right), shrinkage speed (left), rescue frequency (top) and catastrophe frequency (bottom). Data are plotted for a temperature-sensitive dynein mutant Dyn2ts and the respective control strain at permissive (22°C) and restrictive temperature (29°C) [89]. (**C**) Microtubule labelling using the ensconsin microtubule domain fused to three tandem copies of GFP [49] results in higher signal noise than direct tubulin labelling in the same cell line as shown in (A). Measurement of microtubule dynamics by recording the length at different time points is indicated in the zoomed section for one growing microtubule. (**D**) Example of an hTERT RPE1 cell expressing EB3-tdTomato that has been analysed using plusTipTracker [79]. Tracks are presented colour-coded indicating average speed, and according to directionality relative to long axis of the cell [90].

Direct labelling of microtubules can generally be achieved by fusing a fluorescent protein (FP) to the N-terminus of α-tubulin or the C-terminus of β-tubulin (Figure 3A,B) [42–44]. While it is not possible to replace all endogenous tubulin with FP fusions due to steric hindrance, endogenous tagging of tubulin has been successfully achieved in mammalian cells [45,46] as these express several isoforms of both α- and β-tubulin and thus only a subset of the dimers in the microtubule will carry a label. An alternative is the use of small organic fluorophores. These can be conjugated to purified tubulin and then introduced by microinjection [39,40] or directly labelled in living cells using genetic code expansion and cell permeable fluorescent dyes such as silicon rhodamine [47]. The latter is an especially powerful approach as the dye attachment site can be chosen freely and thus avoid locations for protein–protein interaction and post-translational modifications. Due to the relatively high cytoplasmic pool of tubulin, an increased SNR can often be obtained when labelling a MAP or small molecule rather than tubulin directly. Lattice-decorating MAPs such as MAP4 and MAP7/ensconsin are commonly used to achieve this (Figure 3C) [48–52]. As many fluorescent fusions of MAPs retain full function, endogenous tagging avoids perturbing microtubule dynamics as may occur with tubulin or MAP overexpression [53]. The docetaxel-based cell-permeable dye SiR-tubulin is a popular alternative for hard-to-transfect cells. At concentrations up to 100 nM, it has been shown to have little effect on cell proliferation and it’s binding on the inside of the microtubule makes it particularly suitable for superresolution techniques [41].

A popular alternative to labelling the entire microtubule is to use tip tracking proteins (+TIPs) such as EB1, EB3 or CLIP-170 that track the growing microtubule end (Figure 3D) [54,55]. EBs allow the reliable measurement of microtubule assembly even within dense microtubule structures and are the marker of choice to measure microtubule nucleation rate from specific locations within the cell and to determine the polarity of microtubules (Figure 3D) [7,56–61]. However, +TIPs only provide insights into microtubule assembly and it remains challenging to measure other aspects of microtubule dynamics in dense arrays such as the mitotic spindle or in neurites. One approach to measure microtubule turnover is to use photoactivatable or convertible FP fusions to tubulin and then measure the dissipation of the activated proteins from a region of interest [62]. Alternatively, the incorporation time of single tubulin subunits can be directly measured in sparsely labelled samples [63]. In addition, both methods provide information on the displacement of microtubules.

In most cells, microtubules grow at an average rate of 0.2–0.4 µm/s and shrink substantially faster. Thus to not miss rapid shortening events, imaging at 1 frame per second is required. Using widefield or confocal microscopy, usually only one imaging plane can be acquired at this rate without causing photodamage that alters both microtubule dynamics as well as cell morphology. For adherent cells, TIRF microscopy offers an increase in SNR and reduced phototoxicity, but imaging is limited to a few hundred nanometres near the ventral surface of the cell [64]. But the true revolution in live cell imaging of dynamic microtubules is due to lattice light sheet microscopy (LLSM) as it allows the acquisition of entire cell volumes in one second and the thinness of the light sheet enables timelapse imaging at this rate for many minutes without photodamage [65]. LLSM has already been successfully used to quantify EB tracks throughout the entire mitotic spindle [61] and is likely to become the state-of-the art.

Determining the four parameters of dynamic instability in cells is relatively straightforward in cellular systems with very few or spaced out microtubules [43,66–69] (Figure 3B). However, a complication to determining parameters of dynamic instability in cells is the spatial regulation of microtubule dynamics, thus reporting global parameters might not always make sense. In many cells, microtubules grow relatively unperturbed until they reach the cell boundaries and catastrophes and rescues occur primarily near the cell cortex [69,70]. Microtubule assembly – or at least the progress of EB comets – slows down in the proximity of the cell cortex [71], possibly due to increased resistance in the actin-dense lamella and retrograde actin flow [72]. Also, microtubule dynamics change during the cell cycle and some parameters of dynamic instability change up to 4-fold if comparing interphase and mitotic microtubules [73]. Therefore, to obtain insights into the function of a microtubule regulator, it is important to compare similar regions of cells with similar morphology in the same cell cycle state.

## Data analysis and presentation

The most common and most reliable way to extract microtubule life history data is to track the position of the microtubule end or to measure the length of a microtubule manually at every time point [74,75]. To account for changes in the curvature of the microtubule, a curve can be traced or fitted to the entire length of the microtubule [69]. If the entire microtubule is not visible, this can be done from an arbitrary point on the microtubule lattice (Figure 3C). If microtubules do not undergo significant sideways movements, generating kymographs as for *in vitro* data is a faster alternative as it does not require measuring every timepoint individually. An elegant way to visualize microtubule growth and shortening events is sequential subtraction analysis [76]. By subtracting images with a time shift of one or several frames from each other, both regions with polymer gain as well as regions with polymer loss can be calculated and tracked. In cells, in which lateral movement of microtubules is slow in comparison with length changes, this technique allows tracking growth and shrinkage even in dense networks [76,77].

If growing microtubules have been labelled with +TIPs, automatic tracking and analysis of microtubule assembly parameters is a possibility. Using a pattern recognition approach in Labview, EB comets were tracked in *C. elegans* embryos to conduct a screen for factors required for microtubule growth and nucleation [57]. To study microtubule polarity in Drosophila oocytes, limitations of low SNR mainly due to the autofluorescence from yolk were overcome by a combination of probabilistic foreground extraction and adaptive mean filtering to allowed automatic segmentation and tracking of EB foci [78]. PlusTipTracker is a MATLAB-based package for tracking and analysing EB comets or any bright spots based on the knowledge that microtubule trajectories are almost straight [71,79] (Figure 3D). The software package also infers shrinkage rates and rescue frequencies from comets reappearing on the previous trajectory of a comet. In dense microtubule networks, the inferred data are probably not informative. Further, if the data are from a single plane, comets are lost and gained due to growing out or into the focal plane, further complicating any analysis beyond microtubule assembly speed. However, EB tracking performed on timelapse data from the entire cell volume as available from lattice light sheet imaging allows extracting information from birth to catastrophe. In pioneering work, 3D tracking of EB comets was performed using Imaris followed by post-analysis in MATLAB to determine microtubule assembly properties in different stages of human mitosis [61].

It is common practice to present microtubule dynamics parameters as mean ± SD in a table. A visual alternative are diamond graphs that show the four parameters of dynamic instability and allow easy comparison between different treatments [80] (Figure 3B). However, growth and shrinkage velocities are not normally distributed and it is feasible to expect that some microtubule regulators speed up velocity in the cell body but result in reduced assembly near the cell cortex or vice versa. Such changes to the distribution would be ignored if just reporting mean data for the entire cell. Violin plots (Figure 2C) are a space-saving way to show and compare distributions between different conditions, while speed maps are useful to visualize their spatial distribution (Figure 3C).

## Measuring microtubule nucleation and self-renewal

It is becoming recognized at one of the most important events in the life of a microtubule is its birth. Thus regulating where and when microtubules are nucleated is a key aspect in organizing microtubule arrays. To measure microtubule nucleation rates *in vitro*, counting microtubules formed 15 min after warming the sample or adding free tubulin to a template is a simple and effective approach that would usually be performed for a range of tubulin concentrations [15,81]. Nucleating activity would then be detected by a shift of the curve to lower tubulin concentrations. To determine the sites and frequency of microtubule nucleation in cells, a number of complementary approaches are being used. In microtubule regrowth experiments, all microtubules are depolymerized using either a depolymerizing drug or incubation on ice, before their regrowth is observed from different locations in the cell [59,82]. The experiment works on the assumption that nucleation templates stay in position when microtubules are removed. Newly appearing EB comets can indicate either a microtubule birth or rescue – as long as out of focus entry can be excluded. Especially if comparing nucleation from different subcellular structures such as the centrosome and the Golgi, counting emanating EB tracks is a robust method to determine relative nucleation efficiency [58]. Corroborating evidence can be provided by localizing known microtubule nucleators such as centrosome components, minus end stabilizers of the CAMSAP family, or minus-end directed kinesin fusions such as Nod-KHC [82–84]. An elegant approach to determine the position of microtubule minus ends in the mitotic spindle is to cut microtubules with a laser and determine the extent of microtubule depolymerization [85].

Another emerging topic in the measurement of microtubule dynamics is the exchange of tubulin not at the tip of the microtubule, but along the lattice. Tubulin exchange occurs at sites of microtubule defects that either occur spontaneously during microtubule assembly and correlate with the speed of microtubule polymerization or can be introduced by mechanical stress on the microtubule such as repeated bending [86,87]. Tubulin exchange can be detected by changing the colour of the free tubulin pool. *In vitro*, microfluidics allows the gentle exchange of the solution with tubulin containing a different label [87]. In cells, photoconversion of mEos2-tubulin has been used to the same effect [88]. Spots in the ‘new’ tubulin colour appear at sites of repair after several minutes of incubation. These findings suggest that microtubule repair could result in a significant turnover of tubulin subunits in long-lived microtubules away from the microtubule tips and lattice binding MAPs might have a role in regulating this tubulin exchange mechanism.

## Conclusions

We expect that our knowledge in microtubule regulation will increase dramatically over the next decade due to several recent breakthroughs: (1) Advances in live cell imaging allow 3D timelapse imaging with unprecedented temporal resolution, (2) the cryoEM revolution enables uncovering the structural changes in tubulin following nucleotide hydrolysis and the interactions of microtubule regulators with tubulin and (3) the purification of recombinant human tubulin enables studying point mutations, modifications and isoforms. Therefore, establishing robust assays to measure microtubule dynamics both *in vitro* and in living cells will be important to push the boundary of our understanding. The biggest challenges for *in vitro* experiments are the use of buffers that might not be physiologically relevant and a lack of standard in tubulin preparations across both laboratories and prep-to-prep variability. *In vivo*, it is increasingly apparent that different cell types express different compositions of tubulin isoforms, and regulate microtubule dynamics locally and temporally; thus, microtubule dynamics is context dependent. To truly understand how simple tubulin dimers can build the beautiful and complex microtubule network, we must gain context-specific understanding of how tubulin isoforms, tubulin modifications and MAPs guide the fascinating phenomenon of dynamic instability.

## Summary

- The proper regulation of microtubule dynamics is essential for faithful chromosome segregation, cellular morphology and motility.
- Microtubules are long polymers undergoing phases of continuous growth and shrinkage.
- The four parameters of dynamic instability typically measured are growth speed, shrinkage speed, catastrophe rate and rescue rate.
- Microtubule dynamics can be reconstituted *in vitro*, which is useful to measure the direct effect of individual proteins or small molecules.
- A number of strategies are available to label microtubules in cells to observe and measure their dynamics in their physiological context.

## Abbreviations

CFP: cyan fluorescent protein
DIC: differential interference contrast
EB: end-binding protein
FP: fluorescent protein
GFP: green fluorescent protein
GMPCPP: Guanosine-5′-[(α,β)-methyleno]triphosphate
LLSM: lattice light sheet microscopy
MAP: microtubule-associated protein
RPE: retinal pigment epithelium
SiR: silicon rhodamine
SNR: signal–noise ratio
+TIP: tip tracking protein
TIRF: total internal reflection fluorescence
YFP: yellow fluorescent protein
